# Exosomes and the Prion Protein: More than One Truth

**DOI:** 10.3389/fnins.2017.00194

**Published:** 2017-04-19

**Authors:** Alexander Hartmann, Christiane Muth, Oliver Dabrowski, Susanne Krasemann, Markus Glatzel

**Affiliations:** ^1^Center of Diagnostics, Institute of Neuropathology, University Medical Center Hamburg-EppendorfHamburg, Germany; ^2^Center for Applied Nanotechnology, GmbHHamburg, Germany

**Keywords:** prion, exosome, micro vesicle, extracellular vesicle, neurodegeneration, beta-amyloid, prion disease, Alzheimer's disease

## Abstract

Exosomes are involved in the progression of neurodegenerative diseases. The cellular prion protein (PrP^C^) is highly expressed on exosomes. In neurodegenerative diseases, PrP^C^ has at least two functions: It is the substrate for the generation of pathological prion protein (PrP^Sc^), a key player in the pathophysiology of prion diseases. On the other hand, it binds neurotoxic amyloid-beta (Aß) oligomers, which are associated with initiation and progression of Alzheimer's disease (AD). This has direct consequences for the role of exosomal expressed PrP^C^. In prion diseases, exosomal PrP leads to efficient dissemination of pathological prion protein, thus promoting spreading and transmission of the disease. In AD, exosomal PrP^C^ can bind and detoxify Aß oligomers thus acting protective. In both scenarios, assessment of the state of PrP^C^ on exosomes derived from blood or cerebrospinal fluid (CSF) may be useful for diagnostic workup of these diseases. This review sums up current knowledge of the role of exosomal PrP^C^ on different aspects of Alzheimer's and prion disease.

## Introduction

Due to demographic shift in modern societies, neurodegenerative diseases are increasingly prevalent mainly among the elderly population (Prince et al., 2013). The progressive and irreversible degeneration of synapses and neurons leading to nervous system dysfunction is a hallmark of these diseases. In the vast majority of neurodegenerative conditions neuronal decay associates with generation and aggregation of specific proteins in the brain, thus these diseases are termed cerebral proteinopathies (Jellinger, 2003). A rare, yet well studied example of this group of diseases are prion diseases, where misfolding and deposition of PrP^C^ into its pathogenic counterpart PrP^Sc^ plays a key role in disease initiation and progression (Prusiner, 1982; Aguzzi and Lakkaraju, 2016). Alzheimer's disease, on the other hand, represents a highly prevalent example of a cerebral proteinopathy (Walsh and Selkoe, 2016). Here, generation and deposition of aggregation-prone Aß peptide is involved in disease pathogenesis. The two diseases are linked not only by a considerable overlap regarding clinical presentation but also by shared risk genes and shared molecular pathways underlying neurodegeneration (Uchiyama et al., 2013; Udayar et al., 2013). Accordingly, it was not surprising to see that PrP^C^ specifically binds ß-sheet rich proteins such as aggregated Aß or aggregated PrP^Sc^ (Lauren et al., 2009; Resenberger et al., 2011; Um et al., 2012; Falker et al., 2016). Consequences of this binding are less clear with some studies identifying PrP^C^ as a high affinity receptor for oligomeric Aß transducing neurotoxic signaling (Walsh et al., 2002; Lesne et al., 2006), whereas others favor a role of this binding in clearance of neurotoxic proteins (Pflanzner et al., 2012). Furthermore, PrP^C^ participates in Aß transcytosis across the blood-brain barrier (Devraj et al., 2016).

How misfolded protein species spread from neuron to neuron in the central nervous system (CNS) in neurodegenerative diseases is a matter of debate. Direct cell-cell contact might play a role, but in recent years other mechanisms such as vesicular transport have been proposed (Kalani et al., 2014). Exosomes are small extracellular vesicles that may facilitate spreading of disease pathology in dementia (Fevrier et al., 2004). Neuronal exosomes are highly enriched in PrP^C^. Because of the multiple roles of PrP in neurodegenerative diseases, a closer look is warranted. Thus, here we focus on the multiple roles that exosomal PrP^C^ might play in the pathophysiology of neurodegenerative diseases.

## The cellular prion protein (PrP^C^)

The membrane-associated glycoprotein PrP^C^ is involved in diverse processes including neuronal differentiation, neuroprotection, signal transduction, and cell adhesion (Vassallo and Herms, 2003; Aguzzi and Lakkaraju, 2016). It is highly expressed on neurons and present to a lower extent on other cell types such as lymphoid cells and myocytes (Vassallo and Herms, 2003; Aguzzi and Lakkaraju, 2016). The prion protein is composed of an unstructured N-terminal domain and a globular structured C-terminus, comprising mostly alpha-helices. PrP^C^ is C-terminally anchored to the outer leaflet of the plasma membrane via its glycosylphosphatidylinositol (GPI) anchor and positioned in lipid rafts (Riek et al., 1997; Biasini et al., 2012).

In prion diseases, PrP^C^ is converted into a disease-associated isoform of itself termed PrP^Sc^. Seeds of multiple PrP^Sc^-molecules promote further conversion of PrP^C^ into PrP^Sc^ in a self-propagating mechanism thereby multiplying the amount of PrP^Sc^ and leading to aggregation and deposition of PrP^Sc^ in the brain (Telling et al., 1996). Whereas PrP^C^ displays an alpha-helical structure, PrP^Sc^ is richer in beta-sheets. The latter renders PrP^Sc^ more stable against proteolytic digestion. Its partial resistance to proteinase K digestion is used as a diagnostic tool in prion diseases. However, it should be noted that protease sensitive prion species exist and that the relation between PK-resistant PrP^Sc^ and prion infectivity is not linear (Manson et al., 1999; Krasemann et al., 2013). A single amino acid alteration (101L) introduced into murine PrP dramatically alters incubation time of transmissible spongiform encephalopathy (Manson et al., 1999) and it is hypothesized that more than one prion species exist.

## Exosomes in physiology

Exosomes are small membranous vesicles found in a variety of body fluids and the extracellular space, with a diameter ranging from 50 to 150 nm (Raposo et al., 2011). They are generated by invagination of endosomal membranes to form intraluminal vesicles (ILVs) within multivesicular bodies (MVBs). Sorting of ILVs to lysosomes leads to degradation, whereas fusion of MVBs with the plasma membrane and subsequent exocytosis leads to release of these extracellular vesicles (Raposo et al., 2011; Guo et al., 2016). Thus, the distinction of exosomes from other extracellular vesicles such as microvesicles, which directly bud from the plasma membrane, is not trivial with an obvious morphological overlap between the two. Nevertheless, for terms of clarity and to facilitate integration of existing literature, in this review we will use the term “exosomes” to describe a subset of small extracellular vesicles defined by their size, their separation on sucrose density gradients, their protein composition and their shape (Falker et al., 2016). Most commonly, differential centrifugation is used for isolating exosomes from cell culture media or body fluids (Leblanc et al., 2017). Most of the papers cited in this article used modified versions of this ultracentrifugation protocol for exosome isolation. Further characterization using marker proteins for vesicles derived from intracellular multivesicular bodies (CD9, CD63, CD81, and TSG101) are used to ensure presence of exosomes. However, relative ratios of these “exosomal markers” differ between exosomes derived from different cell types (Haraszti et al., 2016) and even further purification with sucrose gradient does not lead to uniform vesicle populations regarding size and marker profile (Bobrie et al., 2012). Thus, it is prudent to point out that extracellular vesicles isolated using current protocols for exosome-isolation, lead to vesicle populations, which are enriched in exosomes, but certainly also contain vesicles of non-exosomal origin (Bobrie et al., 2012; Lotvall et al., 2014; Chernyshev et al., 2015; Kreimer et al., 2015; Abramowicz et al., 2016; Gardiner et al., 2016; Leblanc et al., 2017).

Exosomes transfer cargo such as proteins, lipids and nucleic acids from donor to recipient cells over long distances in a seemingly targeted fashion (Yanez-Mo et al., 2015). This is why they may play important roles in intercellular communication in CNS, where they might mediate neuronal and glia communication (Kramer-Albers and Hill, 2016), promote neuronal repair and growth, regulate the immune response and present antigens (Fröhlich et al., 2014; Yanez-Mo et al., 2015; Guo et al., 2016).

The protein signature of exosomes with a relative overrepresentation of plasma membrane proteins, cytosolic proteins and proteins involved in vesicle trafficking reflects their membranous origin. Interestingly, although PrP^*C*^ is not considered a marker for exosomes, it is highly expressed on these and seems to be actively sorted into exosomes (Hill et al., 2008; Vella et al., 2008a; Falker et al., 2016).

## The role of exosomes in neurodegeneration

Exosomes are of interest in neurodegenerative disease for a number of reasons (Table 1). Firstly, due to their release into the extracellular space, they are attractive targets for diagnostic procedures (Liu et al., 2014; Goetzl et al., 2015).

**Table 1:** **Comparison of the function of exosomal PrP in different diseases**.

	**Negative role**	**Positive role**
Alzheimer's disease	Lipid rafts as sites for initial Aß deposition (Kokubo et al., 2005)	Sequester toxic Aß-oligomers rescuing LTP impairment (An et al., 2013)
	Spreading of toxic Aß-oligomers (Rajendran et al., 2006)	Decreasing Aß levels and deposition *in vivo*, reduction in Aß pathology (Yuyama et al., 2014)
	Exosomal proteins associated to plaques in AD patients brains (Rajendran et al., 2006)	Neuroprotection due to binding and neutralizing of neurotoxic Aß-oligomers (Falker et al., 2016)
Prion disease	PrP^Sc^ as surface protein (Fevrier et al., 2004)	
	Transferring prion infectivity (Fevrier et al., 2004)	
	Facilitate intercellular prion transmission (Guo et al., 2016)	

Furthermore, exosomes play key roles in the pathophysiology of neurodegenerative diseases. Obviously, involvement of exosomes has been documented in prion-diseases such as Creutzfeldt-Jakob disease but also other more common forms of dementias such as Huntington-disease, tauopathies, amyotrophic lateral sclerosis, and Alzheimer's disease (Rajendran et al., 2006; Saa et al., 2014; Asai et al., 2015; Jeon et al., 2016; Polanco et al., 2016; Silverman et al., 2016).

## Exosomal PrP in the pathophysiology of prion disease: spreading the disease

Although there is only limited research on the role of exosomal PrP in prion disease, it is assumed, that exosomal PrP^*Sc*^ promotes dissemination of the disease within the CNS and in the lymphoreticular system.

The first studies suggesting an involvement of exosomal PrP in prion diseases came from cell culture based studies, which revealed an association between exosomes and PrP^Sc^ in media of prion infected cells (Fevrier et al., 2004). Further studies showed a link between release of retrovirus particles, presence of PrP^Sc^, and prion infectivity on both, exosomes and retroviral particles, thus it was proposed that retroviral infection could be a cofactor in the spreading of prion disease (Alais et al., 2012). However, subsequent *in vivo* studies by our group and others did not provide evidence for this, but rather showed that subclinical retroviral infection acts as a disease modifier, but does not enhance spreading of the disease (Alais et al., 2012; Krasemann et al., 2012; Muth et al., 2016).

Further studies focusing on release of PrP^Sc^ from cells not only provided evidence that PrP^*Sc*^ associates with exosomes but also showed that release of PrP^Sc^ and prion infectivity could be attenuated by interfering with exosome biogenesis through inhibition of the endosomal sorting complex required for transport (ESCRT; Alais et al., 2008; Vilette et al., 2015). In line with this study, further research showed that pharmacological stimulation of exosome release by treatment with the ionophore Monensin increased release of infectious exosomes. Moreover, exosomes isolated from these *in vitro* experiments were able to transmit prion disease *in vivo* (Guo et al., 2016). The group of Vella described similar effects *in vitro* and *in vivo*. They showed that exosomes from prion-infected neuronal cell lines are capable to initiate prion propagation in uninfected non-neuronal cells and that these exosomes also provoke prion disease after inoculation in mice (Vella et al., 2007). In peripheral tissues, exosomes are released from prion infected dendritic cells possibly explaining the rapid colonization of prions in the lymphoreticular system (Klohn et al., 2013).

Further evidence for a role of exosomes in transmitting prion disease came from studies on blood-derived exosomes from prion-infected mice. Here, it was shown that those exosomes contained prion infectivity and injection into animals lead to successful transmission of disease (Cervenakova et al., 2016).

## Exosomal PrP in the diagnosis of prion disease: potentially useful

Human prion diseases show a wide spectrum of clinical presentations with disease durations ranging from months to several years (Geissen et al., 2007). Specific isoforms of PrP^Sc^ are related to specific human prion strains, which show differences in deposition pattern and disease manifestation (Wadsworth and Collinge, 2011). Since the definite diagnosis of human prion disease is only possible in a post mortem exanimation, current protocols to establish a probable diagnosis involve assessment of medical history, clinical symptoms and auxiliary tests such as analysis of body fluids e.g., cerebrospinal fluid (Glatzel et al., 2005; Collins et al., 2006). With current approaches assessing markers of neuronal death as surrogates for prion-caused neurodegeneration it is not possible to differentiate between prion strains. Recent data showed differences in the sorting of different prion strains into exosomes (Arellano-Anaya et al., 2015). Additionally, new techniques enable detection of minimal amounts of exosome associated PrP^Sc^ in blood (Berrone et al., 2015; Properzi et al., 2015). Thus, it is possible that the function of exosomes to spread prion infectivity and PrP^Sc^ in the body, may in fact be advantageous if exploited in a diagnostic setting.

## Exosomal PrP in the pathophysiology of AD: neurotoxicity vs. protection

In AD, Aß-oligomers rather than Aß aggregates cause synaptic dysfunction and network failure (Lesne et al., 2006). In advanced AD stages the Aß-oligomer equilibrium in the brain is shifted to more fibrillary Aß occurring as Aß-plaques. Nevertheless, there are still soluble oligomeric forms of Aß present, potentially leading to neurotoxicity.

In 2005 it was described that membrane-bound Aß is associated with lipid rafts within senile plaques, suggesting rafts as sites for initial Aß deposition (Kokubo et al., 2005). Hereon, it could be shown that exosomal proteins likewise accumulated in plaques of AD patient brains, suggesting a role of exosomes in AD (Rajendran et al., 2006). Interestingly, the inhibition of exosome secretion in an AD mouse model resulted in the reduction of Aß and Aß plaque formation, suggesting a disease promoting role for exosomes in AD (Dinkins et al., 2014).

In contrast, exosomes have been shown to induce Aß aggregation into non-toxic fibrils thus decreasing synaptotoxicity by clearance of potentially neurotoxic small Aß-oligomers in the extracellular space, possibly by enabling more efficient Aß uptake into microglia (Yuyama et al., 2012, 2014). In order to achieve these effects, Aß has to associate with exosomes. In principle, exosomes may be directly capable to process the amyloid precursor protein into Aß peptides (Vella et al., 2008b). However, it is more likely that cell derived Aß binds to exosomes. How this binding is achieved is a matter of debate and will be discussed below.

Exosomes have been shown to bind toxic Aß-oligomers and favor accelerated conversion into nontoxic Aß-fibrils resulting in protection from Aß-oligomer induced toxicity (Yuyama et al., 2012). We found that exosomal PrP^C^ specifically binds to Aß-oligomers (Falker et al., 2016). Of note, binding of Aß-oligomers to neuronal PrP^C^ is known to cause synaptic dysfunction (Lauren et al., 2009; Benilova et al., 2012). Thus, our finding opens up new facets for the Aß-receptor hypothesis and may explain why the enrichment of PrP^C^ on exosomes correlates with the ability to sequester Aß-oligomers. Highest binding affinities to cell membrane PrP^C^ where shown for small Aß_42_ species (dimers to pentamers) representing the major neurotoxic Aß-entities in AD (Lauren et al., 2009; Benilova et al., 2012). Binding of these Aß-species to exosomal PrP^C^ resulted in fibrillization of neurotoxic Aß-entities into non-neurotoxic Aß-fibrils (Falker et al., 2016). The association of Aß and exosomes in the context of AD was also described in several publications by the group of Igaraschi. They showed that exosomes injected to the brain of AD mice associated with Aß which resulted in reduction of Aß pathology. Based on their findings they suggested that exosomes released from brain cells play a role in modulating Aß metabolism (Yuyama et al., 2014). The main difference to our studies is that Yuyama et al. suggested glycosphingolipids (GSLs) as the binding partner of Aß oligomers which are also enriched on exosomes (Yuyama et al., 2015), whereas we could identify PrP^C^ on exosomes as a modulator of Aß binding and maybe detoxification (Falker et al., 2016). Since both data sets convincingly showed reduction of Aß toxicity, exosomal PrP^C^ and GSLs may have complementary functions.

**Figure 1:**
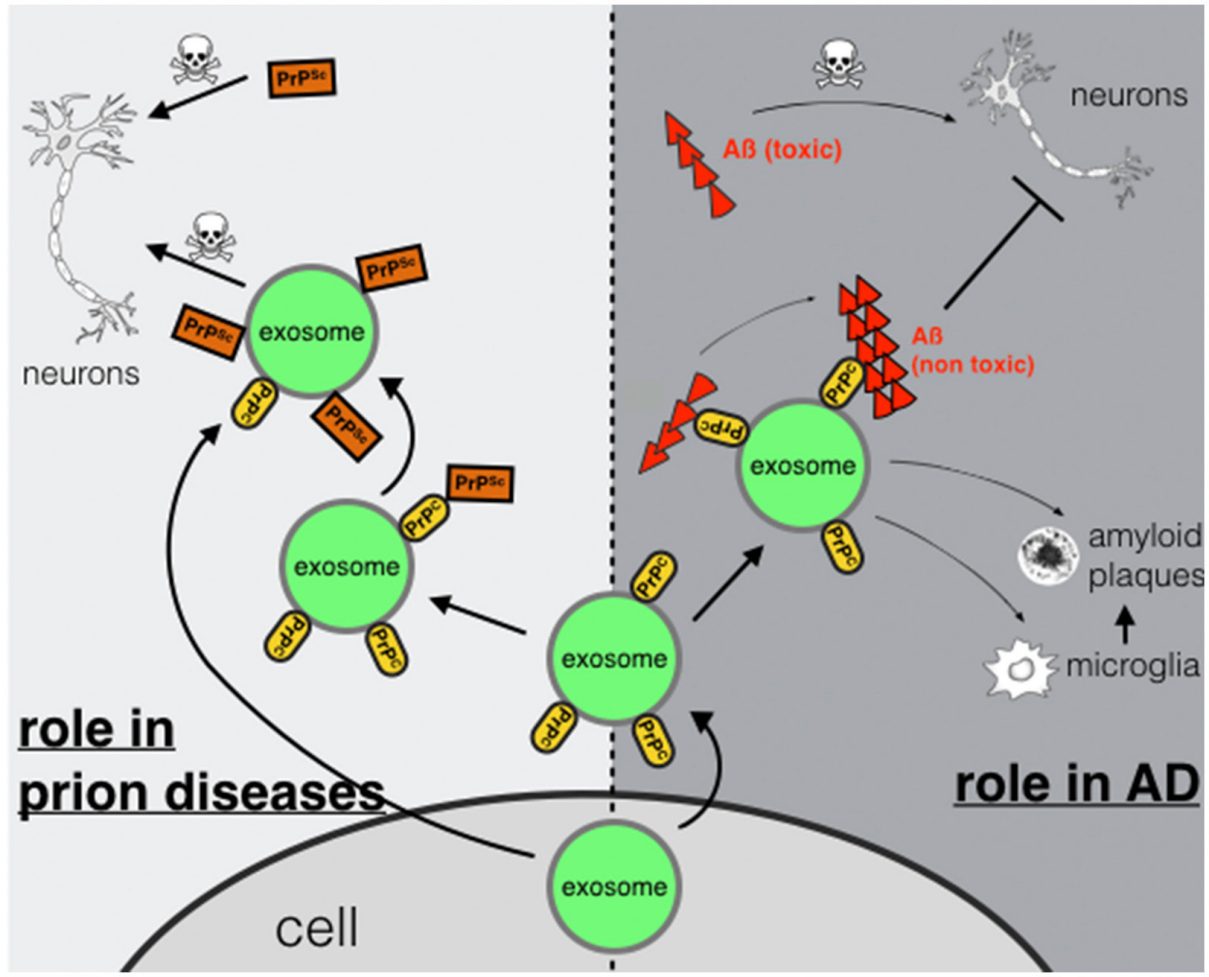
**Exosomal PrP^C^: spreading or trapping of neurotoxic proteins in neurodegeneration**. Role in prion diseases **(left)**: transport of PrP^Sc^ via exosomes secreted from a prion-infected cell or binding of PrP^Sc^ to exosomal PrP^C^ may enhance transmission and spreading. Role in AD **(right)**: capturing and detoxifying of neurotoxic Aß-peptides by exosomal PrP^*C*^ may act neuroprotective. Aß-fibrils bound to exosomes may contribute to Aß plaque formation or may enable uptake and degradation by microglia.

In conclusion we suggest a dual role for PrP^C^ in AD. On the one hand, binding of Aß-oligomers to PrP^C^ on neuronal plasma membranes may act neurotoxic possibly by inducing downstream neurotoxic signaling cascades (Figure 1). On the other hand, PrP^C^ present on membranes of exosomes may act neuroprotective by accelerating conversion to non-toxic Aß-species. If this binding leads to enhanced degradation by microglia cells (An et al., 2013) or promotes deposition of Aß in non-toxic aggregation states such as-plaques (Rajendran et al., 2006) remains to be studied in more detail.

## The future of exosomes and dementia

Studying the functions of PrP^C^ on exosomes in neurodegenerative diseases is tedious. This is in part due to the small size and extreme mobility of exosomes. The reliable tracking of exosomes allowing to determine the fate of exosomes on the cellular level is indispensable for this line of research. Current exosome labeling methods are not stable enough (e.g., pkh-membrane labeling dyes) or demand difficult pre-analytical steps (e.g., WGA-conjugates, transfection). New approaches generating persistent labels on exosomes without altering their properties would open up new perspectives in this line of research.

Furthermore, exosomes may be used in therapeutic contexts with the aim to deliver cargo specifically to the CNS due to their ability to cross the blood brain barrier (Record et al., 2011; Tominaga et al., 2015). To this respect, recent data revealed a possibility to target specific cellular populations e.g., in the brain, using exosomes to deliver therapeutic reagents (Alvarez-Erviti et al., 2011; Liu et al., 2015).

## Conclusion

Exosomal PrP has at least two functions in neurodegenerative diseases. In prion diseases, the role of exosomal PrP is mostly disease-promoting, by spreading PrP^Sc^ and transferring prion infectivity. In AD, protective effects may be predominant, by sequestering toxic Aß-oligomers in the extracellular space leading to reduced neurotoxicity. Exosomal PrP^*C*^ plays a central role in both aspects, as visualized in Figure 1 and summerized in Table 1. Further, work into the mechanism of this dual role is warranted. For this, novel reliable tools for exosome tracking at cellular level *in vitro* and *in vivo* are required.

## Author contributions

All authors listed, have made substantial, direct and intellectual contribution to the work, and approved it for publication.

### Conflict of interest statement

The authors declare that the research was conducted in the absence of any commercial or financial relationships that could be construed as a potential conflict of interest.

## Abbreviations

PrP: prion protein
PrP^C^: cellular prion protein
PrP^Sc^: scrapie prion protein
Aß: amyloid-beta
AD: Alzheimer's disease
CNS: central nervous system
GPI: glycosylphosphatidylinositol
nm: nanometer
ILVs: intraluminal vesicles
MVBs: multivesicular bodies
e.g.: for example
ESCRT: endosomal sorting complex required for transport
vs: versus
GSLs: glycosphingolipids
PKH: Paul Karl Horan
WGA: Wheat Germ Aggluthinin
kDA: Kilodalton
GFP: green fluorescent protein
CSF: cerebrospinal fluid.
